# Activating Nrf2 signalling alleviates osteoarthritis development by inhibiting inflammasome activation

**DOI:** 10.1111/jcmm.15905

**Published:** 2020-09-23

**Authors:** Zijian Yan, Weihui Qi, Jingdi Zhan, Zeng Lin, Jian Lin, Xinghe Xue, Xiaoyun Pan, Yulong Zhou

**Affiliations:** ^1^ Department of Orthopaedic The Second Affiliated Hospital and Yuying Children’s Hospital of Wenzhou Medical University Wenzhou China; ^2^ Key Laboratory of Orthopaedics of Zhejiang Province Wenzhou China; ^3^ The Second School of Medicine Wenzhou Medical University Wenzhou China

**Keywords:** chondrocyte, Licochalcone A, NLRP3 inflammasome, osteoarthritis, pyroptosis

## Abstract

Osteoarthritis (OA), which is characterized by proliferation of subchondral bone and the degeneration of articular cartilage, is the most prevalent human arthritis. Nod‐like receptor pyrin domain 3 (NLRP3) inflammasome is a hot spot in recent year and has been reported to be associated with OA synovial inflammation. However, there are few studies on NLRP3 inflammasome in chondrocyte. Licochalcone A (Lico A), a compound extracted from Glycyrrhiza species, has various biological effects such as anti‐inflammation, anti‐apoptotic, anti‐cancer and anti‐oxidation. In this study, we investigated the protective effect of Lico A on chondrocytes stimulated by lipopolysaccharide (LPS) and surgically induced OA models. In vitro, Lico A could reduce the expression of NLRP3, apoptosis‐associated speck‐like protein (ASC), Gasdermin D (GSDMD), caspase‐1, interleukin‐1beta (IL‐1β) and IL‐18, which indicated that Lico A attenuates LPS‐induced chondrocytes pyroptosis. In addition, Lico A ameliorates the degradation of extracellular matrix (ECM) by enhancing the expression of aggrecan and collagen‐II. Meanwhile, we found that Lico A inhibits NLRP3 inflammasome via nuclear factor erythroid‐2‐related factor 2 (Nrf2)/haeme oxygenase‐1(HO‐1)/nuclear factor kappa‐B (NF‐κB) axis. And the Nrf2 small interfering RNA (siRNA) could reverse the anti‐pyroptosis effects of Lico A in mouse OA chondrocytes. In vivo, Lico A mitigates progression OA in a mouse model and reduces OA Research Society International (OARSI) scores. Thus, Lico A may have therapeutic potential in OA.

## 1 INTRODUCTION

Osteoarthritis (OA) is the most prevalent arthritis in elderly aged individuals, which hallmark is proliferation of subchondral bone and the degeneration of articular cartilage, resulting in the loss of joint function.^1^ Millions of people around the world suffer from this disease with consequent disability.^2^ Although the specific mechanisms that cause OA are unknown, growing evidence has revealed that catabolic alterations and inflammation plays a momentous role in the development of OA.^3^ It is reported that OA is a low‐grade chronic inflammatory condition and various inflammatory cytokines play a vital role in OA.^4^ Interleukin‐1beta (IL‐1β) and IL‐18, as inflammatory factors, are well established to enhance catabolic effects in the pathogenesis of OA, which could facilitate the degradation of major extracellular matrix (ECM) by inhibiting the production of proteoglycans, aggrecan and type II collagen.^5^

Pyroptosis, a new type of caspase‐1–dependent programmed cell death, has been shown in recent study.^6^ As one of inflammasomes, nod‐like receptor pyrin domain 3 (NLRP3) is a protein complex composed of NLRP3, apoptosis‐associated speck‐like protein(ASC) and caspase‐1 that can be activated by lipopolysaccharide (LPS), which is responsible for regulating inflammatory response.^7^, ^8^, ^9^ Then, the activation of inflammasome can activate and cleave caspase‐1 to trigger the maturation and secretion of IL‑1β and IL‐18, thus regulating inflammation.^10^ Particularly, it has been shown that NLRP3 exerts an inflammatory role in the development of OA.^11^ Meanwhile, nuclear factor kappa‐B (NF‐κB) pathway is a prerequisite for activating NLRP3 inflammasome.^7^ In the early stage of the development of OA, non‐steroidal anti‐inflammatory drugs (NSAID) are considered to be the most effective drugs for the treatment, accompanied by a series of serious side effects.^12^ Therefore, there is an urgent need for a safe and effective drug to alleviate cartilage degeneration and development of OA and inhibiting NLRP3 inflammasome may provide new treatments for OA.

Licochalcone A (Lico A), a phenolic compound extracted from the roots of Glycyrrhiza species, has been reported to have various pharmacological properties, including anti‐inflammation, anti‐apoptotic, anti‐cancer and anti‐oxidation.^13^, ^14^, ^15^, ^16^ Pervious study has shown that Lico A inhibits LPS‐mediated inflammation by blocking NF‐κB activation.^17^ Also, Lico A has been found to attenuate LPS‐induced hepatotoxicity by activating nuclear factor erythroid‐2–related factor 2 (Nrf2).^18^ Meanwhile, a recent study found that Lico A could suppress NLRP3 inflammasome to attenuate acne symptoms.^19^ However, presently, it is not clear whether Lico A attenuates chondrocyte NLRP3 inflammasome, pyroptosis and OA development in vivo. To the best of our knowledge, in this study, we first to show the anti‐inflammasome effect of Lico A in chondrocytes. Thus, we hypothesized that LPS‐induced inflammasome associated IL‑1β and IL‐18 overproduction results in pyroptosis of chondrocytes, consequently promoting the progress of OA. However, Lico A may decrease chondrocytes pyroptosis via the Nrf2/HO‐1/NF‐κB axis.

## 2 MATERIALS AND METHODS

### 2.1 Reagents

Lico A (purity > 98%) was acquired from Beijing Solarbio Science &Technology Co, Ltd (Beijing, China). Cell‐Counting Kit‐8 (CCK‐8) assays were purchased from Dojindo (Kumamo, Japan). Primary antibodies directed against aggrecan, collagen‐II, IκBα, NLRP3,ASC,pro‐caspase 1 and Lamin B were obtained from Abcam (Cambridge, MA, USA). Type II collagenase, LPS and dimethylsulphoxide (DMSO) were acquired from Sigma Chemical Co. (St. Louis, MO, USA). Primary antibodies against Gasdermin D (GSDMD), cleaved‐GSDMD, Nrf2, HO‐1, cleaved caspase‐1, glyceraldehyde‐3‐phosphate dehydrogenase (GADPH) were acquired from Cell Signaling Technology (MA, USA). Alexa Fluor®594‐labelled, Alexa Fluor®488‐labelled goat anti‐rabbit IgG (H + L) secondary antibody and Goat anti‐rabbit IgG‐HRP were was obtained from Jackson ImmunoResearch (West Grove, PA, USA). Enzyme‐linked immunosorbent assay (ELISA) kits were obtained from R&D systems (Minneapolis, MN, USA). Foetal bovine serum (FBS) and Dulbecco's modified Eagle's medium (DMEM)/Ham's F12 medium were purchased from Gibco (Grand Island, NY, USA).

### 2.2 Animals

Forty‐five 8‐week‐old C57BL/6 male mice were acquired from the Animal Center of Wenzhou Medical University. The protocols for animal care and use were adhered to the Guide for the Care and Use of Laboratory Animals of the National Institutes of Health and were authorized by Wenzhou Medical University Animal Care and Use Committee. All mice were anesthetized by intraperitoneal injection of 2% (w/v) pentobarbital (40 mg/kg). The OA model was established with the surgical destabilization of the medial meniscus (DMM) as previously described.^20^ The tibial ligament of the medial meniscus of the right knee joint of each mouse was exposed through the incision of the right medial articular capsule and transected with a micro scalpel. Also, the right knee joints of the mice were underwent an arthrotomy without transection of the medial collateral ligament in the sham control group. Finally, allocating forty‐five mice into three groups randomly with 15 mice in each group: sham control group (sham), OA group (DMM) and Lico A‐treated OA group (DMM + Lico A).

### 2.3 Histological analysis

Eight weeks after surgery, mouse knee joint specimens were rinsed and fixed in 4% paraformaldehyde (Sigma Chemical Co.) for 24 hours and then transferred to 10% EDTA solution (Solarbio Science & Technology) for 4 weeks, with replacing the solution every three days. Finally, the specimens were dehydrated then embedded in paraffin. 5 μm sections and 10 slides per joint were stained with Safranin O/fast green stains (S‐O staining). Depletion of proteoglycan and degeneration of cartilage were conducted using the OA Research Society International (OARSI) system.^21^ Histomorphology was evaluated on 15 mice in each group.

### 2.4 Air pouch mouse model

Before establishing the air pouch model, the C57BL/6 mice were anesthetized by intraperitoneal injection of 2% (w/v) pentobarbital (40 mg/kg). A 5 mL aseptic air was injected into the back to establish a subcutaneous airbag, and 3 mL aseptic air was injected again 3 days later. On day 7, the animals received 1 mL of NS or LPS (1 µg/mL) into the air pouch. And 1 mL of Lico A (10 mg/kg) or NS were injected 1 hour later. After 6 hours, the fluids of air pouch were collected. The fluids were centrifuged with 13 800 *g* for 5 minutes and analysed for IL‐1β and IL‐18 by ELISA.

### 2.5 Primary mouse chondrocyte culture

The chondrocytes was isolated from knee cartilage of male C57BL/6 mice (within 10 days of birth, Animal Center of the Chinese Academy of Sciences). The articular cartilage tissues were collected and rinsed with PBS three times and thereafter incubated with 6‐10 mL of 2 mg/mL (0.1%) type II collagenase for 4 hours at 37°C. The detached cells were centrifuged at 1000 rpm for 3 minutes and transferred to the culture flask for incubate in DMEM/F‐12 supplemented with 10% FBS and 1% antibiotics (penicillin/streptomycin) in a humidified atmosphere containing 5% CO_2_ at 37°C. The culture medium was changed every day and the cells passaged when 80% to 90% confluent, after using 0.25% trypsin‐EDTA solution. Only passage 0 to 2 cells were used to avoid phenotype loss in this study.

### 2.6 CCK‐8 assay

CCK‐8 assay was used to evaluate the underlying toxicity of Lico A on chondrocytes. Briefly, chondrocytes were inoculated in the wells of a 96‐well plate at a density of 8000/well with varying concentrations (0, 5, 25, 50 and 100 μmol/L) for 24 and 48 hours. Then, the cells were incubated with 10 μL of CCK‐8 solution for an additional 4 hours at 37°C. The optical densities of the samples were measured at 450 nm on a spectrophotometer (Leica Microsystems, Germany).

### 2.7 ELISAs

ELISA kits (R&D Systems, Minneapolis, MN, USA) were used to analyse the secretion of IL‐1β, IL‐18, aggrecan and collagen‐II into the culture medium.

### 2.8 LDH release assay

LDH release assay was used to assay LPS‐induced cytotoxicity. Chondrocytes were seed into 96‐well plates and treated with LPS (1 μg/mL) and then incubated with different concentrations of Lico A (5‐50 μmol/L) for 24 hours in the incubator maintained at 5% CO_2_ at 37°C. Cell culture medium was collected and LDH activity was measured, in accordance with the manufacturer's instructions (Beyotime, Nanjing, China).

### 2.9 Western blotting

Proteins of mouse chondrocytes were extracted using radio immunoprecipitation assay (RIPA) lysis buffer, confused with 1% PMSF (phenylmethanesulphonyl fluoride). Enriched protein concentration in each sample was analysed using a bicinchoninic acid assay (BCA) protein assay kit (Beyotime.CN). Cell protein (30 ng) was loaded onto sodium dodecyl sulphate‐polyacrylamide gel electrophoresis (SDS‐PAGE) and then transferred to polyvinylidene difluoride membranes (PVDF). 5% non‐fat dried milk was used to block non‐specific proteins for 60‐120 minutes in room temperature, and then, the membranes were immersed in the primary antibodies against Nrf2 (1:1000), HO‐1 (1:1000), NLRP3 (1:1000), Lamin B (1:1000), ASC (1:1000), pro‐caspase1 (1:1000), cleaved‐Caspase1 (1:1000), GSDMD (1:1000), cleaved‐GSDMD (1:1000), IκBα (1:1,000), p65 (1:1000), aggrecan (1:1000), collagen‐II (1:1000) and GADPH (1:5000) at 4°C then followed by treated with a secondary antibody (1:3000) for 2 hours in room temperature. The protein blots were detected using electrochemiluminescence plus reagent (Invitrogen) the next day. Finally, we used to quantify the intensity of the blots by using Image Lab 3.0 software (Bio‐Rad).

### 2.10 Immunofluorescence microscopy

Chondrocytes were seeded on glass coverslips in a 6‐well plate and cultured for 24 hours. The cells were treated with or without LPS (1 μg/mL) and 50 µmol/L Lico A for 6 hours and then following treatment of ATP (5 mmol/L) for 30 minutes after incubation overnight with serum‐starved medium. The chondrocytes were fixed in 4% paraformaldehyde, then permeated with 0.1% Triton X‐100 (Solarbio Science & Technology), blocked with 10% goat serum, and then incubated overnight with primary antibodies as follows: Nrf2 (1:200), ASC(1:200), p65 (1:200), collagen‐II (1:200) in a humid chamber at 4°C. The next day, the cells were then incubated with Alexa Fluor^®^488 or Fluor^®^594‐conjugated secondary antibody (1:400) for 45 minutes then labelled with DAPI (Solarbio Science & Technology) in the dark for 60 seconds. Images were captured using fluorescence microscopy (Olympus Inc, Tokyo, Japan). The blind study of the experimental group was carried out with ImageJ software 2.1 (NIH, Bethesda, MD, USA) to evaluate the fluorescence intensity.

### 2.11 TUNEL assay

A TUNEL Assay Kit was used to observe the degree of chondrocyte pyroptosis. Chondrocytes were seeded on glass coverslips in a 6‐well plate and cultured for 24 hours. The cultured chondrocytes were fixed with 4% paraformaldehyde for 20 minutes, incubated with 3% H_2_O_2_ and 0.1% Triton X‐100 for 10 minutes, stained with in situ cell death detection kit (Hoffmann‐La Roche Ltd., Basel, Switzerland) and finally labelled with DAPI for 1 minute. Images were captured using fluorescence microscopy (Olympus Inc, Tokyo, Japan).

### 2.12 Real‐time Quantitative PCR (RT‐qPCR)

In accordance with the manufacturer's instructions, the total RNA of chondrocytes stimulated with LPS (1 μg/mL), ATP(5 mmol/L) and Lico A at different concentrations was isolated by using TRIzol reagent (Invitrogen). The synthesis of cDNA is accomplished by using 1000 ng of total RNA (MBI Fermantas, Germany). The conditions of RT‐qPCR were as follows: 95°C for 10 minutes, followed by 40 cycles of 95°C for 15 seconds and 60°C for 60 seconds. A total volume of 10 μL, containing 5 µL SYBR Master Mix, 4.5 μL diluted cDNA, 0.25 μL forward primer and 0.25 μL reverse primer. The reaction was carried out using CFX96 real‐time qPCR system (Bio‐Rad Lab, California, USA). The cycle threshold (Ct) values were normalized to the level of GAPDH. And the levels of each target gene were analysed. The primers of IL‐1β, IL‐18, ASC, NLRP3, caspase1, GSDMD which were listed as follows: IL‐1β: (F) 5′‐CTTCAGGCAGGCAGTATCACTC‐3′; (R)5′‐TGCAGTTGTCTAATGGGAACGT‐3′; IL‐18: (F)5′‐GCCTCAAACCTTCCAAATCA‐3′; (R) 5′‐TGGATCCATTTCCTCAAAGG‐3′; ASC: (F)5′‐GACAGTGCAACTGCGAGAAG‐3′; (R) 5′‐CGACTCCAGATAGTAGCTGACAA‐3′; NLRP3:(F)5′AGCCTTCCAGGATCCTCTTC‐3′; (R) 5′‐CTTGGGCAGCAGTTTCTTTC‐3′; Caspase‐1: (F) :5′‐ACACGTCTTGCCCTCATTATCT‐3′; (R) 5′‐ATAACCTTGGGCTTGTCTTTCA‐3′; GSDMD: (F): 5′‐CCAACATCTCAGGGCCCCAT‐3′; (R) 5′TGGCAAGTTTCTGCCCTGGA‐3′.

### 2.13 Small interfering RNA (siRNA) transfection

Nrf2 siRNA was purchased from Invitrogen (Carlsbad, CA, USA) with the specific following sequences synthesized: sense, 5′‐UUGGGAUUCACGCAUAGGAGCACUG‐3′; antisense, 5′‐CAGUGCUCCUAUGCGGAAUCCCAA‐3′. Transfection of negative control (NC) siRNA and Nrf2 into chondrocytes with Lipofectamine 2000 siRNA transfection reagent (Thermo Fisher, UT, USA), in accordance with the manufacturer's instructions. After further treatment, cells were harvested for Western blot analysis.

### 2.14 Statistical analysis

The data were at least repeated three times. The values are expressed as the mean ± standard deviation (SD). Statistical Product and Service Solutions (SPSS) version 20.0 software was used to performed statistical analyses. Kruskal‐Wallis and One‐way analysis of variance (ANOVA) and Kruskal‐Wallis tests were generated for multiple group comparisons. The differences between groups would be regarded as significant when *P*‐value below 0.05.

## 3 RESULTS

### 3.1 Cytotoxic effect of Lico A on mouse chondrocytes

**Figure 1:**
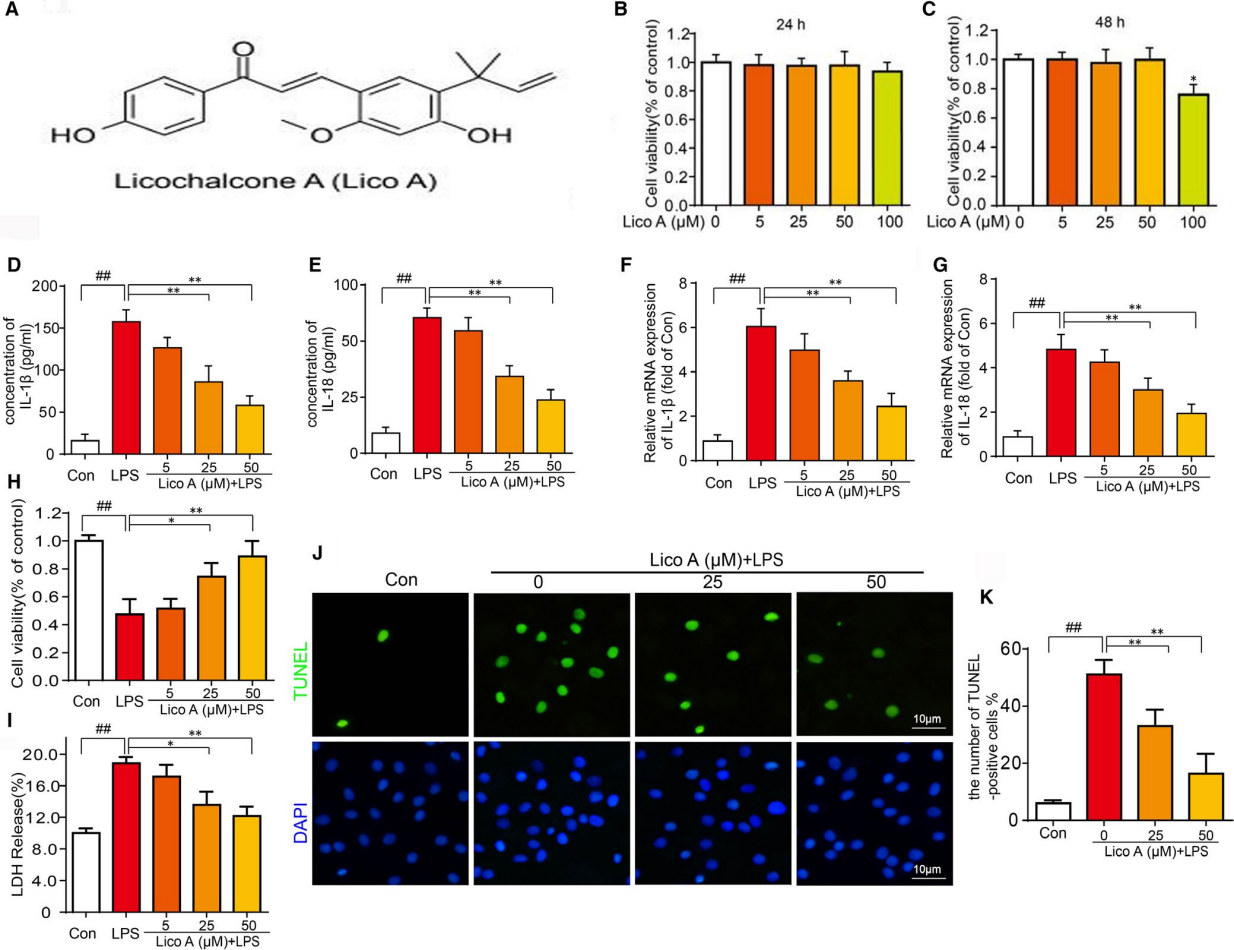
Effects of Lico A on the cell cytotoxicity in vitro and Lico A attenuate LPS‐induced IL‑1β and IL‐18 secretion and cell death. Chemical structure of Lico A (A). The cytotoxic effects of Lico A on chondrocytes were determined with increasing concentrations (0, 5, 25, 50 and 100 μmol/L) for 24 and 48 h using a CCK8 assay (B‐C). The levels of IL‐1β and IL‐18 treated as above determined were by ELISAs (D‐E). The mRNA expression of IL‑1β and IL‐18 were measured by real‐time qPCR (F‐G). The viability of chondrocytes induced by LPS was determined by LDH release and CCK8 assay (H‐I). And TUNEL assay was performed in chondrocyte as described above (J‐K). Values represent the averages ± SD Significant differences between different groups are indicated as ^##^ *P* < 0.01, vs control group; *P < 0.05, **P < 0.01, vs LPS alone treatment group, n = 5

The chemical structure of Lico A is shown in Figure 1A. First, we assessed the cytotoxic effect of Lico A on chondrocyte. The cells were cultured with Lico A at different concentrations (0, 5, 25, 50 and 100 μmol/L) for 24 and 48 hours. Finally, CCK‐8 analysis was used to measure its effect on cell cytotoxicity. As shown in Figure 1B,C, no apparent cytotoxic effects on chondrocytes were observed after concentrations of 5, 25, 50 μmol/L at either 24 or 48 hours.

### 3.2 Lico A attenuates LPS‐induced IL‑1β and IL‐18 secretion and cell death

To further investigate the effect of Lico A on the production of IL‐1β and IL‐18 in chondrocytes in response to LPS, ELISAs were performed. We can see from the Figure 1D,E, after LPS treatment, IL‐1β and IL‐18 production in cell suspension increased, while Lico A inhibited LPS‐induced IL‐1β and IL‐18 production in a dose‐dependent manner, which was consistent with the trend of RT‐qPCR (Figure 1F,G).

As shown in Figure 1H,I, the viability of chondrocytes induced by LPS was significantly decreased and the release of LDH was increased. However, Lico A has an obvious protective effect on cell viability and against LDH release assay. And the results of TUNEL assay showed that the TUNEL‐positive cells induced by LPS was increased, and Lico A pre‐treatment could significantly reduce the TUNEL‐positive cells induced by LPS (Figure 1J,K).

### 3.3 Lico A attenuate LPS‐induced NLRP3 inflammasome and pyroptosis

**Figure 2:**
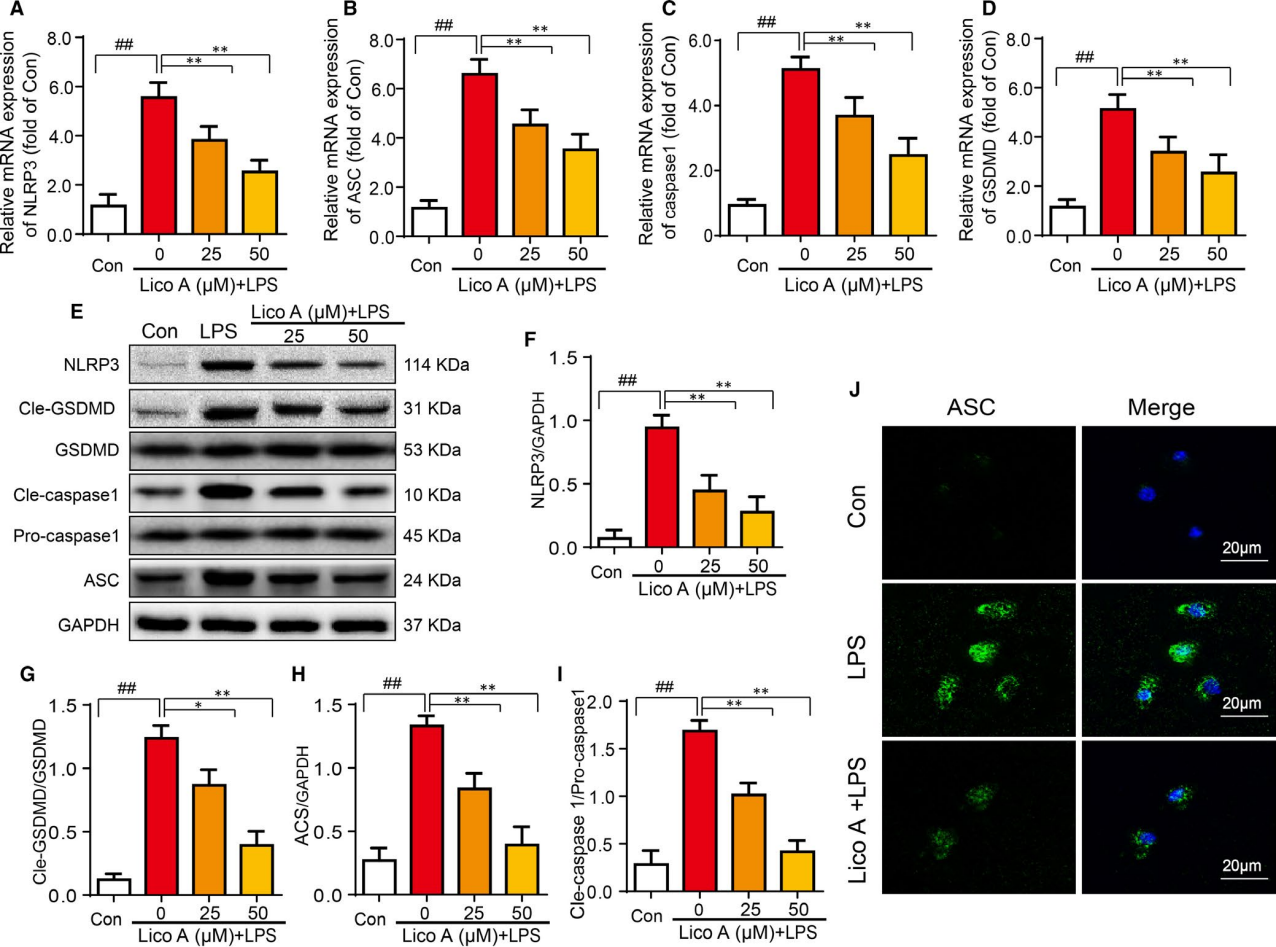
Lico A attenuates LPS‐induced NLRP3 inflammasome and pyroptosis. The chondrocytes were primed with LPS (1 µg/mL) alone or with various concentrations of Lico A together for 6 h, and then following treatment of ATP (5 mmol/L) for 30 min. The mRNA expression of NLRP3, GSDMD, caspase‐1 and ASC in chondrocytes treated as above were visualized by real‐time qPCR (A‐D). The protein expression of NLRP3, GSDMD, cleaved‐GSDMD, pro‐caspase‐1, cleaved caspase‐1 and ASC were measured by Western blot (E‐I). ASC (J) was detected by immunofluorescence combined with DAPI staining. Values represent the averages ± SD Significant differences between different groups are indicated as ^##^ *P* < 0.01 vs control group; *P < 0.05, **P < 0.01 vs LPS alone treatment group, n = 5

To ascertain the potential mechanism of inhibitory effect of Lico A on pyroptosis in chondrocytes, Western blotting, RT‐qPCR and immunofluorescence microscopy were applied. As shown in Figure 2A‐D, the result of RT‐qPCR showed that NLRP3, GSDMD, caspase‐1 and ASC were significantly up‐regulated after treatment with LPS (1 μg/mL). Lico A inhibits the expression of these mediators stimulated by LPS in a dose‐dependent manner, which was consistent with the trend of Western blotting (Figure 2E‐I).

The immunofluorescence results showed that there was almost no expression of ASC in the control group. However, it was obviously expressed in the LPS group. And the fluorescence intensity of ASC of chondrocytes decreased significantly when the chondrocytes were treated with Lico A and LPS (Figure 2J). Above all, the results showed that Lico A attenuate LPS‐induced NLRP3 inflammasome and pyroptosis.

### 3.4 Effect of Lico A on aggrecan and collagen‐II expression in LPS‐activated mouse chondrocytes

**Figure 3:**
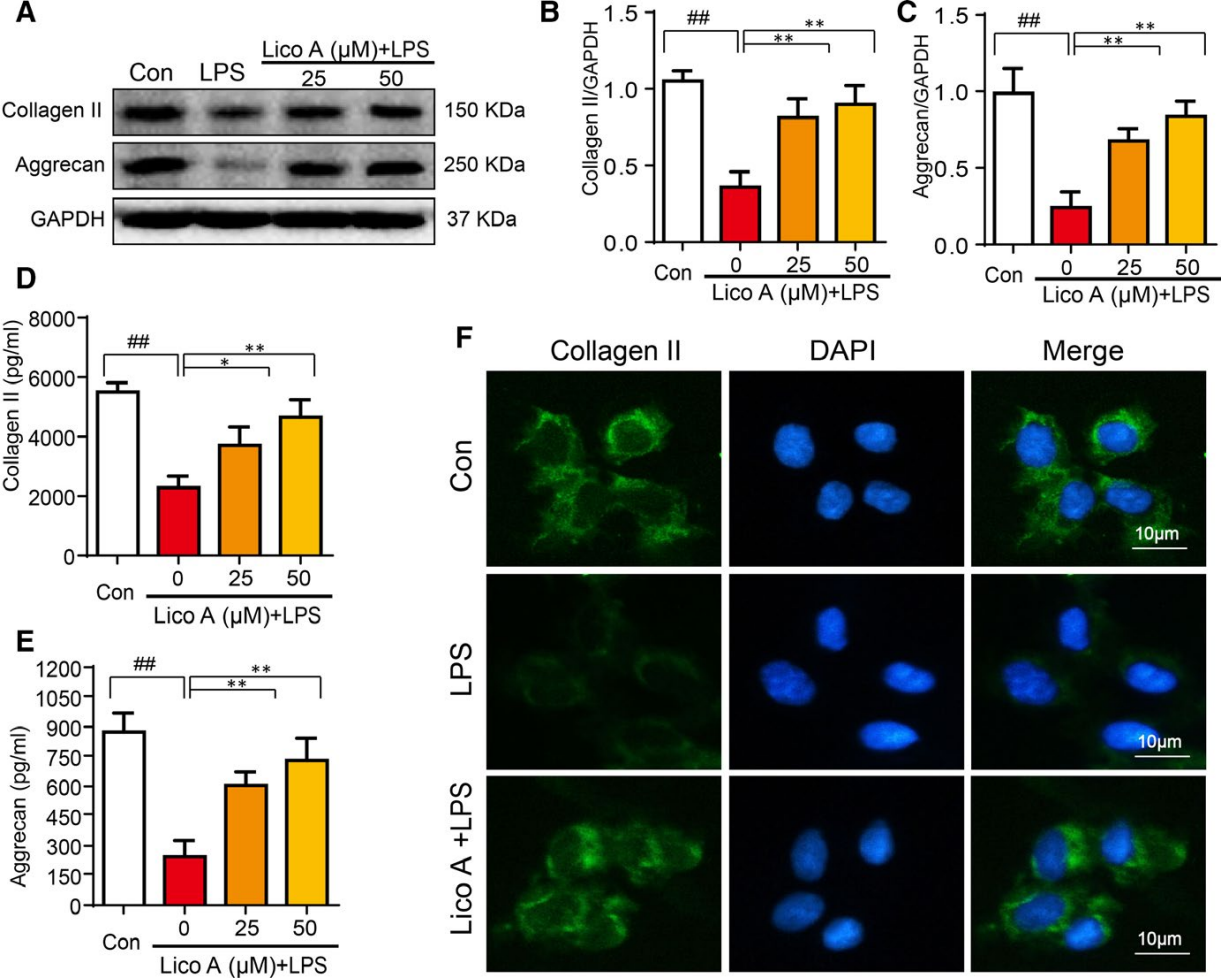
Effect of Lico A on aggrecan and collagen‐II expression in LPS‐activated mouse chondrocytes. The cells were stimulated with LPS (1 µg/mL) alone or by co‐stimulation with LPS (1 µg/mL) and Lico A for 6 h, and then following treatment of ATP (5 mmol/L) for 30 min. The protein expression levels of aggrecan and collagen‐II treated as above were visualized by Western blot (A‐C). The production of aggrecan and collagen‐II in mice chondrocytes were measure by ELISAs (D‐E). Collagen‐II (F) was detected by immunofluorescence combined with DAPI staining. Values represent the averages ± SD Significant differences between different groups are indicated as ^##^ *P* < 0.01, vs control group; *P < 0.05, **P < 0.01, vs LPS alone treatment group, n = 5

Subsequently, to evaluate the role of Lico A in extracellular matrix degradation induced by LPS, we used Western blotting, ELISAs and immunofluorescence analysis. We observed the effects of Lico A on the expression of aggrecan and collagen‐II in chondrocytes. As shown in Figure 3A‐C, LPS considerably reduced the production of aggrecan and collagen‐II. However, Lico A significantly attenuated the inhibitory effect on LPS‐induced protein expression of aggrecan and collagen‐II, which was consistent with the results of ELISAs (Figure 3D,E). Furthermore, immunofluorescence analysis showed that compared with LPS group, Lico A treatment enhanced the production of collagen‐II (Figure 3F).

### 3.5 Effects of Lico A on LPS‐stimulated NF‐κB activation in mouse chondrocytes

**Figure 4:**
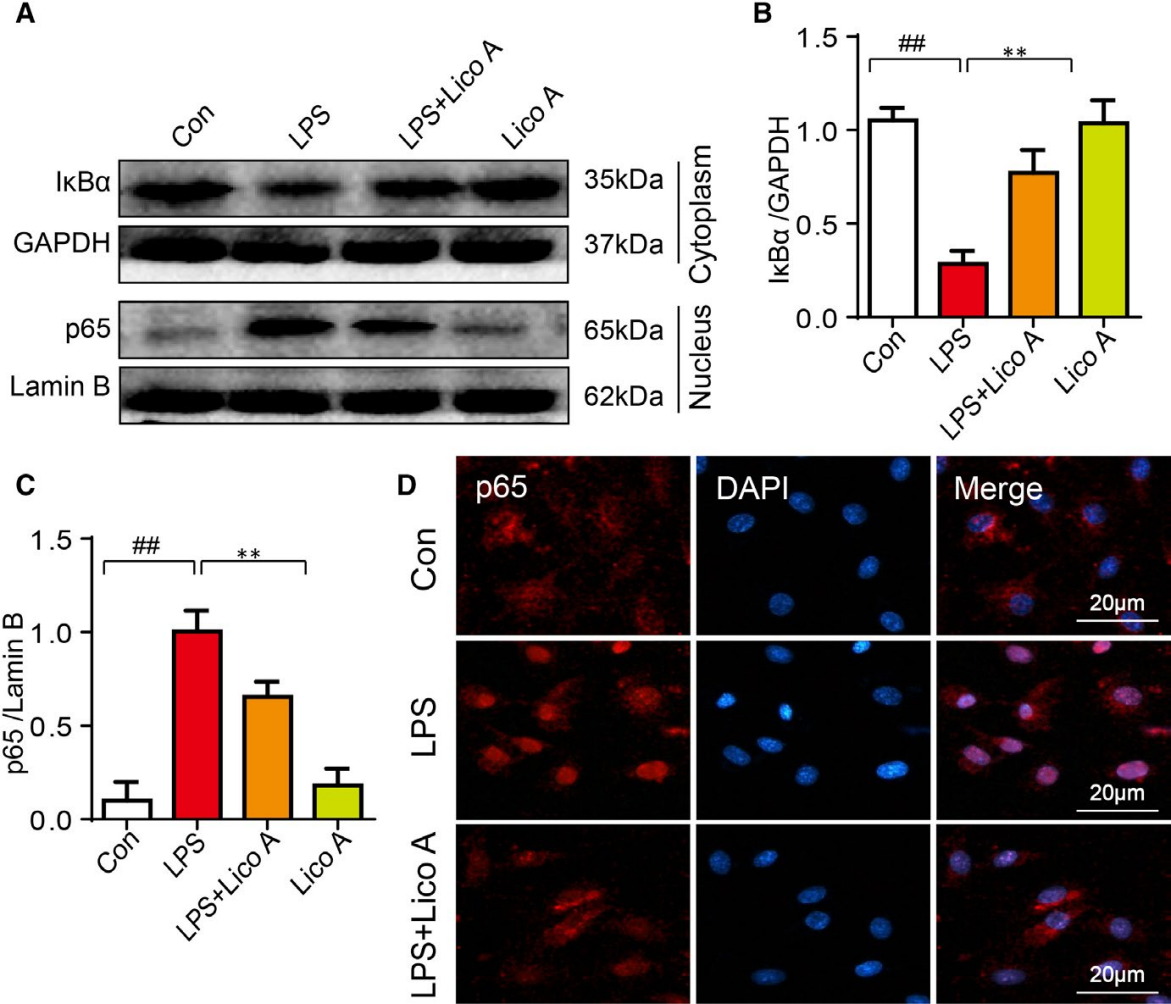
Effects of Lico A on LPS‐stimulated NF‐κB activation in mouse chondrocytes. The chondrocytes were treated with or without LPS (1 μg/mL) and Lico A for 6 h, and then following treatment of ATP (5 mmol/L) for 30 min after incubation overnight. The protein expression levels of p65 and IkBα treated as above were visualized by Western blot (A) and are quantified in (B‐C). The nuclear translocation of p65 was detected by immunofluorescence combined with DAPI staining for nuclei (D).Values represent the averages ± SD Significant differences between different groups are indicated as ^##^ *P* < 0.01, vs control group; **P < 0.01, vs LPS alone treatment group, n = 5

We wished to ascertain the potential mechanism of inhibitory effect of Lico A on NF‐κB pathway in chondrocytes by immunofluorescence microscopy and Western blot analysis. Following 6 hours of LPS and 30 minutes of ATP (5 mmol/L) stimulation, the p65 increased significantly and IκB‐α degraded obviously. However, after treatment with Lico A for 6 hours, the phosphorylation of p65 stimulated by LPS was inhibited, and the degradation of IκBα was also inhibited, and there was no significant difference in the expression of IκBα and p65 between the Lico‐A alone group and the control group (Figure 4A‐C).

In immunofluorescence staining, most of the p65 detected in the control group were concentrated in the cytoplasm, while more in the nucleus in the LPS treatment group. And Lico A pre‐treatment could inhibit the translocation of p65 from cytoplasm to nucleus. (Figure 4D). The above results show that the effect of Lico A is consistent with its inhibitory effect on NF‐κB.

### 3.6 Involvement of Nrf2 in the effect of Lico A on pyroptosis in mouse OA chondrocytes

**Figure 5:**
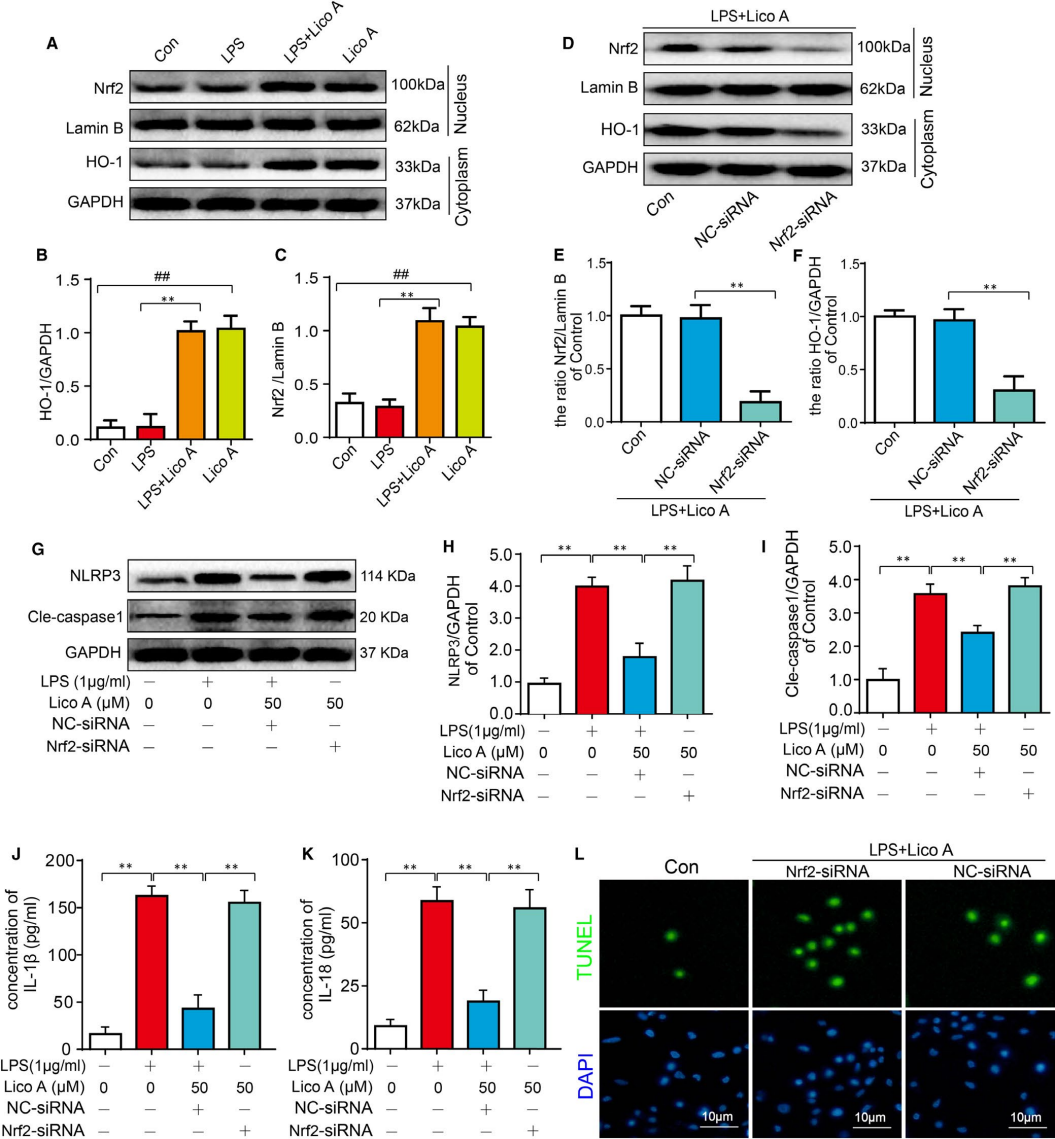
Involvement of Nrf2 in the effect of Lico A on pyroptosis in mouse OA chondrocytes. The protein expression levels of Nrf2 and HO‐1 treated as above were visualized by Western blot (A‐C). After Nrf2 knock down, the protein expressions of Nrf2 and HO‐1 in chondrocytes treated as above were visualized by Western blot (D) and quantified in (E‐F). The protein expressions of NLRP3 and cleaved‐caspase1 in chondrocytes treated as above were visualized by Western blot (G‐I). The production of IL‑1β and IL‐18 in mice chondrocytes treated as above was measured by ELISA (J‐K). And TUNEL assay was performed in chondrocyte as described above (L). Significant differences between different groups are indicated as ^##^ *P* < 0.01, vs control group; **P < 0.01, vs LPS alone treatment group, n = 5

Recent studies revealed that Nrf2/HO‐1 signal pathway might be the key pathway to activate NLRP3 inflammasome. Also, it has been reported that Lico A could activate Nrf2/HO‐1 pathway. However, it is not clear whether Lico A suppresses NLRP3 inflammasome through Nrf2/HO‐1 pathway. Therefore, Western blotting was used to assessLico A clearly increase the expression of Nrf2 and HO‐1 regardless of whether stimulated by LPS or not. However, LPS alone stimulation did not change the expression of Nrf2 and HO‐1 compared to the control group (Figure 5A‐C). To further study whether Lico A exerts its anti‐pyroptosis effect through Nrf2/HO‐1 signal pathway, chondrocytes were transfected with NC‐siRNA and Nrf2‐siRNA. As shown in Figure 5D‐F, Western blot analysis showed that when co‐treated with LPS and Lico A, the expression of HO‐1 and Nrf2 was significantly inhibited after Nrf2 siRNA transfection. And the inhibitory effect of Lico A on NRLP3 and cleaved‐caspase1 elevation could be reversed by Nrf2‐siRNA (Figure 5G‐I). Moreover, we assessed the expression of IL‐1β, IL‐18 by ELISAs and found Nrf2 inhibition blocked the Lico A–mediated anti‐pyroptosis effects (Figure 5J,K), which was consistent with the trend of TUNEL assay (Figure 5L). In summary, it suggested that Lico A exerts its anti‐pyroptosis effect through Nrf2/HO‐1 signal pathway.

### 3.7 Lico A mitigates OA progression and enhance the expression of Nrf2 in a mouse DMM model

**Figure 6:**
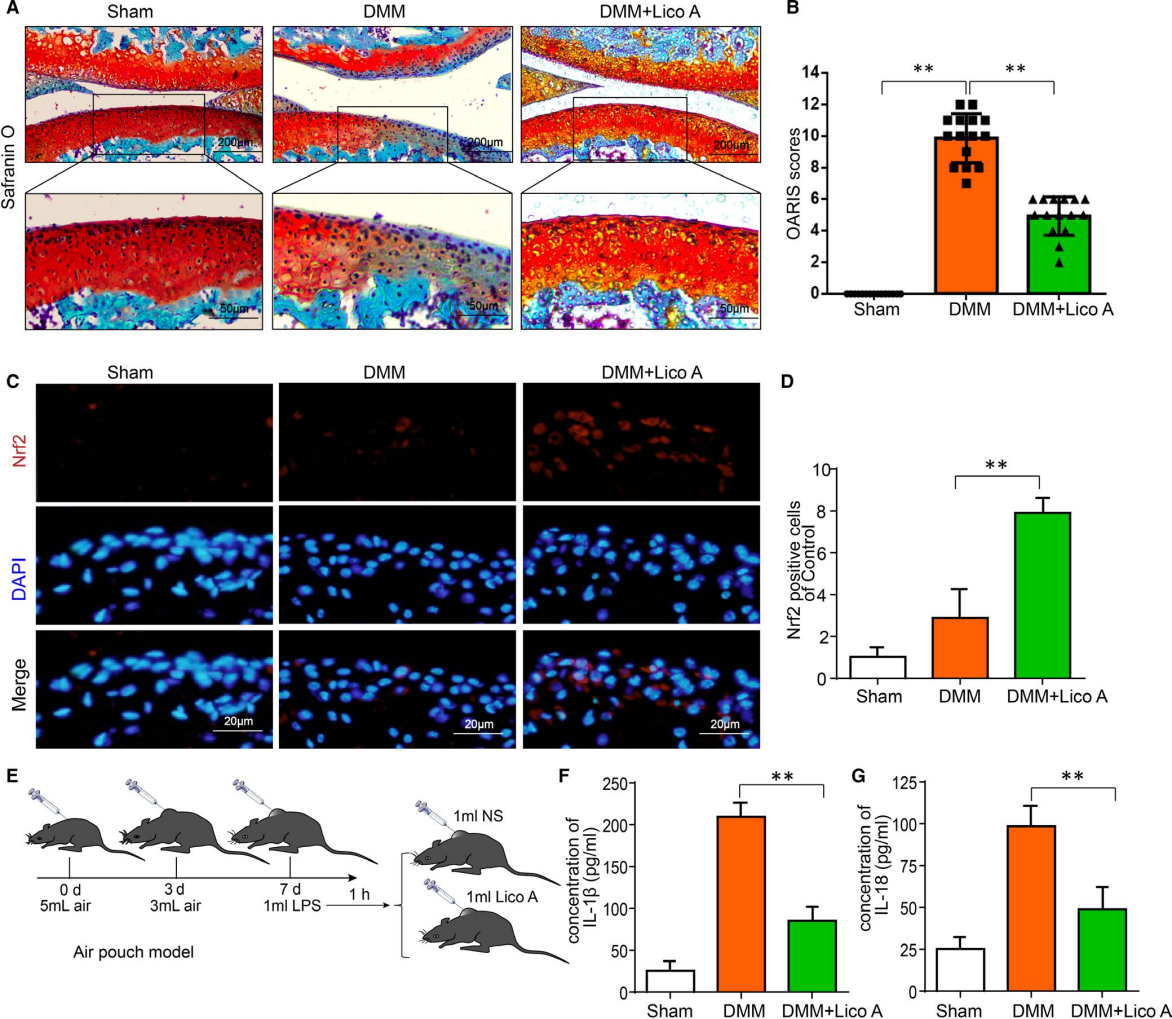
Lico A mitigates OA progression and enhances the expression of Nrf2 in a mouse DMM model. Safranin O staining was used to assess histomorphometric differences between experimental groups at 8 wk post‐surgery (A). Histological analysis of OA was evaluated by Osteoarthritis Research Society International (OARSI) scores (B). Nrf2 (C) was detected by immunofluorescence combined with DAPI staining. The fluorescence intensity of Nrf2 were analysed by ImageJ (D). Air pouch mouse model was used to assess the effect of Lico A on the production of IL‐1β and IL‐18 stimulated by LPS in vivo (E). The production of IL‑1β and IL‐18 in mice chondrocytes treated as above were measure by ELISA (F‐G).Values represent the averages ± SD Significant differences between different groups are indicated as **P < 0.01 vs the Sham group and **P < 0.01 vs the DMM group, n = 15

We established a surgical mouse model of osteoarthritis to study the prophylactic effects of Lico A on the progression of osteoarthritis in vivo, followed by daily 0.5% carboxymethylcellulose (CMC) or intragastric administration of 10 mg/kg Lico A for 8 weeks, which was evaluated by histology of S‐O stained sections. As shown in Figure 6A, the morphology of articular cartilage in the control group was normal and the surface was smooth. While the DMM group exhibited significant hypocellularity, with a rough surface and irregular morphological structure. Conversely, less cartilage erosion, cartilage degradation and proteoglycan depletion were observed in the Lico A treatment group. Furthermore, as presented in Figure 6B, OARSI scores accorded with above S‐O staining results. The OARSI scores of the DMM group were significantly higher than those of the sham control group. However, the OARSI scores were reduced after Lico A treatment. What's more, the tissue sections of the knee joint were stained with Nrf2 by immunofluorescence staining (Figure 6C,D). The expression of Nrf2 was less in sham group and DMM group. However, the number of positive cells increased significantly after treatment with Lico A.

To further study the effect of Lico A on the production of IL‐1β and IL‐18 stimulated by LPS in vivo, air pouch mouse model and ELISAs were carried out. As shown in Figure 6E‐G, production of IL‐1β and IL‐18 was increased in LPS group. However, Lico A significantly attenuated the inhibitory effect on LPS‐induced protein expression of IL‐1 β and IL‐18.

## 4 DISCUSSION

To some extent, the occurrence and development of OA could consider to be related to imbalance between catabolism and anabolism of articular cartilage.^22^ Chondrocytes are the only group of cells in articular cartilage, and their changes play an important role in the process of OA.^23^ Nod‐like receptor pyrin domain 3 (NLRP3) inflammasome is a hot spot in recent study and has been reported to be associated with OA synovial inflammation.^24^ However, there are few studies on NLRP3 inflammasome in chondrocyte and OA. Licochalcone A(Lico A), a compound extracted from Glycyrrhiza species, has various pharmacological properties, including anti‐inflammation, anti‐apoptotic and anti‐oxidation. Therefore, in this study, we have evaluated the protective effect of Lico A on chondrocytes stimulated by LPS and the mechanistic relationship between the inhibitory effect of Lico A on pyroptosis and Nrf2/HO‐1/NF‐κB axis.

It has been proved that caspase‐1 activity in dependent inflammasome can lead to cell death in a highly inflammatory form called pyroptosis.^25^ In the process of pyroptosis, cleave caspase‐1 could trigger the maturation and secretion of IL‑1β and IL‐18 and then expand inflammatory reaction.^26^ And the inhibition of caspase‐1 could slow down the development of chronic arthritis.^27^ IL‑1β, as one of the most prominent inflammation cytokines in OA, has been considered to be implicated in cartilage degradation.^28^, ^29^, ^30^ And several studies have confirmed that IL‐18 participates in the inflammatory response of OA and inhibits the production of aggrecan and collagen‐II.^5^ As an important part of pyroptosis, NLRP3 inflammasome is reported to be involved in the pathogenesis of OA and the expression of NLRP3 protein in patients with knee OA is higher than that of control group.^24^, ^31^ As one of the most widely studied Gasdermin, GSDMD is also involved in pyroptosis.^32^ It could be cleaved by Caspase‐1 to release the N‐terminal domain, which leads to form a pore in the plasma membrane to release substrates, such as IL‐18 and IL‑1β.^33^ And recent study has shown that Loganin could control chondrocyte pyrolysis to reduce ameliorates cartilage damage in osteoarthritis.^34^ In this study, we found that Lico A inhibited the expression of NLRP3, ASC, cleaved‐GSDMD and cleaved‐caspase1 proteins induced by LPS in mouse chondrocytes. What's more, we demonstrated that the significant inhibitory effect of Lico A on the expression of LPS‐induced IL‐18 and IL‑1β in mouse chondrocytes and the blocking effect of Lico A on the production of aggrecan and collagen‐II. Taken together, Lico A was able to suppress LPS‐induced pyroptosis and cartilage degradation by inhibiting NLRP3 inflammasome.

As is known to all, GSDMD‐mediated pyroptosis and activation of NLRP3 inflammasome and requires the involvement of NF‐κB signal transduction pathway, which is necessary for express pro‐IL‐1β and exerts a momentous role in the initiation and assembly of inflammasome.^35^ And previous studies have shown that inflammatory mediators are regulated by NF‐κB signal transduction pathways and participate in the development of OA.^36^, ^37^, ^38^ In the absence of stimulation, the NF‐κB dimers are bound in the cytoplasm by interacting with IκB, an inhibitor of the NF‐κB protein.^39^ LPS has been considered to be a activator of the NF‐κB pathway, which plays a vital role in inflammation.^40^ After stimulation with LPS, IκBα‐p65 binding was phosphorylated, which results in NF‐κB dissociation. Further, the free NF‐κB translocates from the cytoplasm to the nucleus and transactivates various inflammatory factors.^23^, ^39^ In this study, we found that after Lico A treatment, mouse OA chondrocytes reduced the degradation of IκB‐α and the translocation of p65 to the nucleus. These results are demonstrated that Lico A may be involved in the regulation of NLRP3 inflammasome by coordinating NF‐κB pathway in mouse OA chondrocytes.

**Figure 7:**
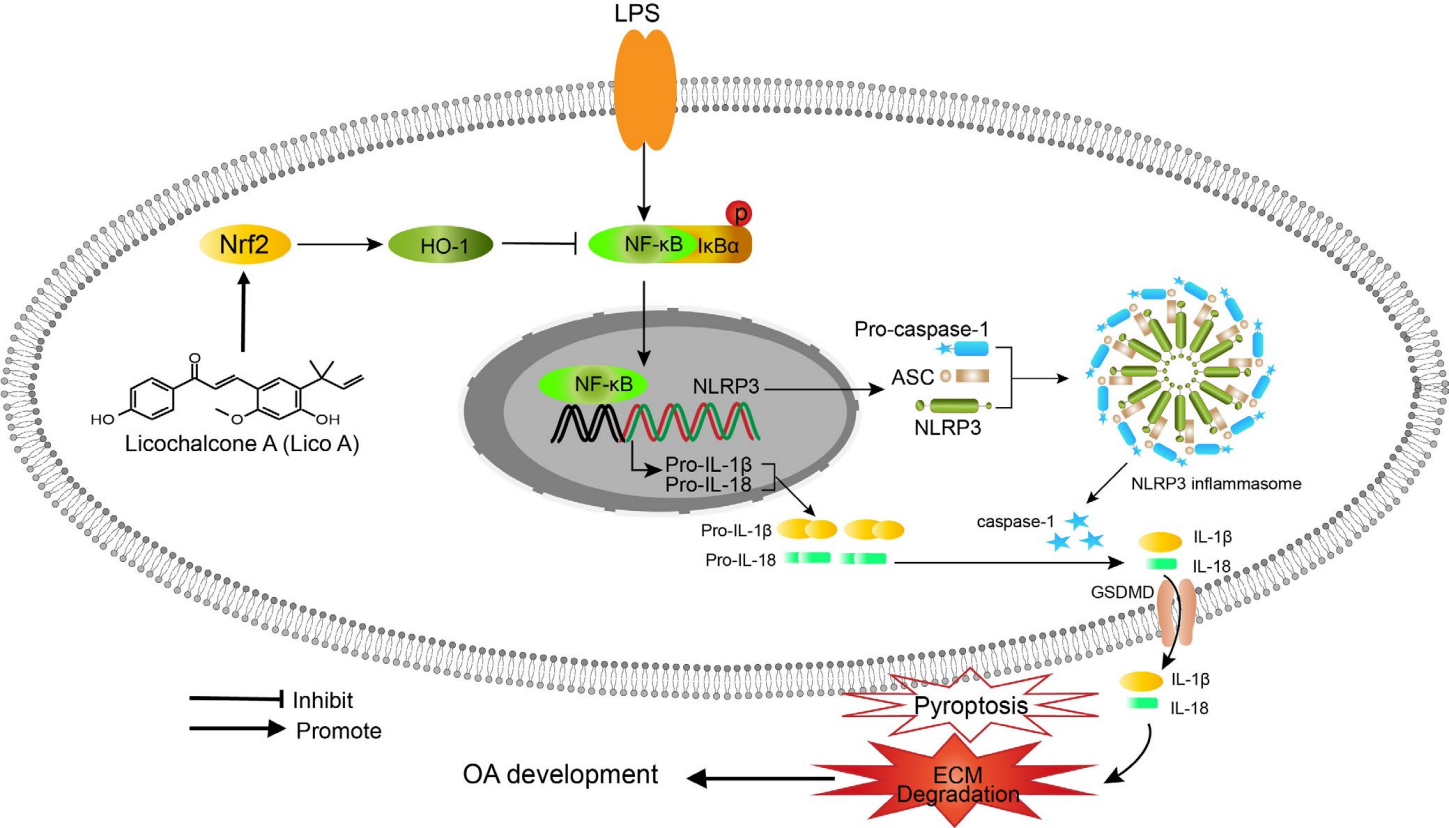
Schematic illustration of the potential protective effects of Lico A in osteoarthritis development

In addition, Nrf2 is a member of the CNC‐bZIP protein family, which's expression mainly located in the cytoplasm.^41^ Nrf2 could transactivate genes driven by antioxidant response element (ARE), especially HO‐1.^42^ The up‐regulated expression of HO‐1 has a protective effect on cells under inflammatory conditions.^43^ Evidence suggests that inhibition of NF‐κB by activating Nrf2/HO‐1 signal pathway could reduce inflammatory response and absence of Nrf2 could increases the production of cytokines through activating NF‐κB pathway, showing an interaction between Nrf2/HO‐1 and NF‐κB pathway.^44^, ^45^ Recent studies revealed that Nrf2/HO‐1 signal pathway might be the key pathway to activate NLRP3 inflammasome and involved in the occurrence and development of OA.^46^ Herein, we speculated that Nrf2, which is activated by Lico A, may exert anti‐pyroptosis effects in OA. In this study, through Lico A‐mediated Nrf2 activation, NLRP3 inflammasome, IL‐18 and IL‑1β were significantly inhibited. However, the absence of Nrf2, which mediated by siRNA, strongly blocked Lico A‐mediated inhibition of LPS‐induced pyroptosis in mouse OA chondrocytes, supporting the role of Nrf2/HO‐1 pathway in the anti‐pyroptosis effect of Lico A (Figure 7).

Furthermore, we also explored the effect of Lico A on OA in vivo. Compared to the sham control group, the DMM group exhibited significant hypocellularity, with a rough surface and irregular morphological structure via S‐O staining. Conversely, Lico A treatment could mitigate these detrimental effects. The conclusions were accorded with above OARSI scores. Meanwhile, the result of air pouch mouse model also demonstrated that Lico A attenuates LPS‐induced pyroptosis in vivo.

In previous study, Lico A could reduce IL‐1β–induced inflammation in chondrocytes via Nrf2 in vitro.^47^ However, in our further research, we found that Nrf2/HO‐1/NF‐κB axis not only participate in IL‐1β–induced inflammation but also in LPS‐induced inflammasome and pyroptosis. Lico A could inhibits NLRP3 inflammasome via Nrf2/HO‐1/NF‐κB axis, which verified by various experimental methods. And the effect of Lico A on OA in vivo has not been demonstrated. Therefore, in our study, Lico A mitigates OA progression in a mouse DMM model and significantly attenuated the increase of LPS‐induced protein expression of IL‐1β and IL‐18 in air pouch mouse model, which further improves the action mechanism of Lico A in OA.

In present study, we demonstrated that Lico A inhibits LPS‐induced NLRP3 inflammasome and pyroptosis through Nrf2/HO‐1/NF‐κB axis. Meanwhile, Lico A treatment ameliorated the OA progression in surgically induced models. Therefore, Lico A may have therapeutic potential in OA.

## CONFLICT OF INTEREST

The authors confirm that there are no conflicts of interest.

## AUTHOR CONTRIBUTIONS

**Zijian Yan:** Data curation (lead); Software (lead); Writing‐original draft (lead); Writing‐review & editing (supporting). **Weihui Qi:** Conceptualization (lead); Methodology (lead); Writing‐review & editing (supporting). **Jingdi Zhan:** Data curation (supporting); Software (supporting); Writing‐original draft (supporting). **Zeng Lin:** Conceptualization (equal); Methodology (equal). **Jian Lin:** Software (supporting); Validation (lead). **Xinghe Xue:** Investigation (supporting); Visualization (supporting). **Xiaoyun Pan:** Supervision (supporting); Writing‐review & editing (supporting). **Yulong Zhou:** Investigation (lead); Supervision (lead); Visualization (lead); Writing‐review & editing (lead).

## Data Availability

The data that support the findings of this study are available from the corresponding author upon reasonable request.
